# Supplementary material for: Closed-loop two-photon functional imaging in a freely moving animal
Source: Nat Commun. 2025 Jul 1;16:5950. doi: 10.1038/s41467-025-60648-x (PMC12214772; doi:10.1038/s41467-025-60648-x)
Supplement: Supplementary file 1 — Supplementary Information [file 41467_2025_60648_MOESM1_ESM.pdf]

Supplement to Closed-loop Two Photon Functional Imaging in a Freely Moving Animal

Paul McNulty[1,†], Rui Wu[1,†], Akihiro Yamaguchi[1], Ellie S. Heckscher[2], Andrew Haas[1], Amajindi Nwankpa[1], Mirna Mihovilovic Skanata[1,*], and Marc Gershow[1,3,4,*]

[1] *Department of Physics, New York University, New York, USA* [2] *Department of Molecular Genetics and Cell Biology, University of Chicago, Chicago, IL, USA* [3] *Center for Neural Science, New York University, New York, USA* [4] *Neuroscience Institute, New York University, New York, USA*

[†] *equal contribution*

[*] *correspondence to mmihovil@syr.edu(MMS), marc.gershow@nyu.edu(MG)*

Supplemental Discussion

Beautiful work in other organisms have used single-photon fluorescence techniques to record neural activity in untethered freely moving animals, including *C. elegans*, larval zebrafish, and hydra [1–11]. But these methods have not translated to larval *Drosophila*, likely due to the complicated motion of the larva which translates and distorts the brain, scattering and autofluorescence of the cuticle whose optical properties change over the course of a crawling stride, and the proximity to the brain of autofluorescent and scattering structures like the gut and trachea.

In the larva, spinning disk confocal microscopy was used to reveal transient bilateral differences in the aggregated activities of 60 neurons driven by thermal activation of ectopically expressed TRPA-1 [12], and to record the temperature responses of peripheral thermosensory neurons in moving larvae during periods of "spontaneous quiescence" [6]. SCAPE microscopy [3] was used to record activity from cuticular proprioceptors [13]; recordings from the same neurons were also achieved using confocal and two-photon tracking microscopy [14]. Both SCAPE [15] and confocal [16] microscopy were used in rolling larvae to record calcium dynamics from a portion of the peripheral *muscles* (not neurons) closest to the microscope objective. To date, recording from individual neurons in the CNS of crawling larva has only been possible using two-photon tracking microscopy [17, 18].

Single-photon and two-photon recordings have been accomplished in behaving animals to which a microscope has been physically attached [19–25]. In mice, wearable miniaturized microscopes have been developed for single-photon [24] and two-photon recordings [21, 25]. In adult *Drosophila*, flies behave in virtual arenas while head-fixed to the microscope [19, 20, 26, 27]. Putting aside questions of the extent to which this tethering affects free behavior, it is not currently possible to mount a microscope on a larva or head-fix a behaving larva, both due to the larva's size and its peristaltic crawling.

Real-time motion correction of two photon microscopy has been a long-standing goal. To combat residual brain motion in head-fixed mice and partially immobilized zebrafish, Griffiths and colleagues [28] developed a motion

correction algorithm based on the tracking of implanted fluorescent beads. This apparatus took advantage of a specially developed 3D acousto-optic lens (AOL) comprised of 4 acousto-optic deflectors [29] (see also [30–34]). Earlier work using an AOL for motion correction without implanted beads [35] was limited to micron-scale corrections. Compared to our approach, the AOL provided the ability to sample arbitrary scan paths, allowing random-access sampling of individual cells at much higher sampling rates (100 Hz) than achieved here. However, our approach does not require the implantation of separate beads for tracking and has been demonstrated to track neurons in fully released animals.

Even were we to implant beads in the larva or otherwise adapt the AOL technology, the random access approach described in [28] would not immediately translate. First, multiple beads or points would need to be tracked to allow for real-time correction of rotation; otherwise it would not be possible to target specific regions for interrogation. Second, due to brain deformation, the sampled volumes would have to be significantly expanded to allow for uncompensated motion of the neurons following rigid body correction. The need to devote more time to tracking and to increase the sampled volumes would decrease the effective sampling rate. If random access scanning is abandoned, the AOL has fewer advantages, because acousto-optics are not particularly fast for volumetric imaging. To allow real-time rotation correction with CRASH2p, we suggest combining 2 AODs with a TAG lens on the tracking path (section 3.2.1, [18]), but an equally attractive approach might be to incorporate an AOL on the tracking path instead.

Supplementary References

1. Faumont, S. *et al.* An Image-Free Opto-Mechanical System for Creating Virtual Environments and Imaging Neuronal Activity in Freely Moving Caenorhabditis elegans. *PLoS ONE* **6,** e24666 (Sept. 2011).
2. Zheng, M., Cao, P., Yang, J., Xu, X. Z. S. & Feng, Z. Calcium imaging of multiple neurons in freely behaving *C. elegans*. *Journal of Neuroscience Methods* **206,** 78–82. ISSN: 0165-0270 (Apr. 2012).
3. Bouchard, M. B. *et al.* Swept confocally-aligned planar excitation (SCAPE) microscopy for high-speed volumetric imaging of behaving organisms. *Nature Photonics* **9,** 113–119. ISSN: 1749-4885, 1749-4893 (Jan. 2015).
4. Kotera, I. *et al.* Pan-neuronal screening in Caenorhabditis elegans reveals asymmetric dynamics of AWC neurons is critical for thermal avoidance behavior. *eLife* **5** (ed Hobert, O.) Publisher: eLife Sciences Publications, Ltd, e19021. ISSN: 2050-084X (Nov. 2016).
5. Nguyen, J. P. *et al.* Whole-brain calcium imaging with cellular resolution in freely behaving Caenorhabditis elegans. en. *Proceedings of the National Academy of Sciences* **113,** E1074–E1081. ISSN: 0027-8424, 1091-6490 (Feb. 2016).
6. Venkatachalam, V. *et al.* Pan-neuronal imaging in roaming Caenorhabditis elegans. en. *Proceedings of the National Academy of Sciences* **113,** E1082–E1088. ISSN: 0027-8424, 1091-6490 (Feb. 2016).
7. Kim, D. H. *et al.* Pan-neuronal calcium imaging with cellular resolution in freely swimming zebrafish. en. *Nature Methods* **14,** 1107–1114. ISSN: 1548-7105 (Nov. 2017).
8. Cong, L. *et al.* Rapid whole brain imaging of neural activity in freely behaving larval zebrafish (Danio rerio). en. *eLife* **6,** e28158. ISSN: 2050-084X (Sept. 2017).
9. Dupre, C. & Yuste, R. Non-overlapping Neural Networks in Hydra vulgaris. English. *Current Biology* **27,** 1085–1097. ISSN: 0960-9822 (Apr. 2017).
10. Kramer, T. S. & Flavell, S. W. Building and integrating brain-wide maps of nervous system function in invertebrates. *Current Opinion in Neurobiology* **86,** 102868. ISSN: 0959-4388 (June 2024).
11. Atanas, A. A. *et al.* Brain-wide representations of behavior spanning multiple timescales and states in C. elegans. English. *Cell* **186.** Publisher: Elsevier, 4134–4151.e31. ISSN: 0092-8674, 1097-4172 (Sept. 2023).
12. Heckscher, E. *et al.* Even-Skipped+ Interneurons Are Core Components of a Sensorimotor Circuit that Maintains Left-Right Symmetric Muscle Contraction Amplitude. *Neuron* **88,** 314–329. ISSN: 0896-6273 (Oct. 2015).
13. Vaadia, R. D. *et al.* Characterization of Proprioceptive System Dynamics in Behaving Drosophila Larvae Using High-Speed Volumetric Microscopy. *Current Biology* **29,** 935–944.e4. ISSN: 0960-9822 (Mar. 2019).
14. He, L. *et al.* Direction Selectivity in Drosophila Proprioceptors Requires the Mechanosensory Channel Tmc. English. *Current Biology* **0.** ISSN: 0960-9822 (Mar. 2019).
15. Cooney, P. C. *et al.* Neuromuscular basis of Drosophila larval rolling escape behavior. *Proceedings of the National Academy of Sciences* **120.** Publisher: Proceedings of the National Academy of Sciences, e2303641120 (Dec. 2023).

16. He, L., Borjon, L. & Tracey, W. D. *The motor pattern of rolling escape locomotion in Drosophila larvae* en. Pages: 2022.11.03.514605 Section: New Results. Nov. 2022.

17. Karagyozov, D., Mihovilovic Skanata, M., Lesar, A. & Gershow, M. Recording Neural Activity in Unrestrained Animals with Three-Dimensional Tracking Two-Photon Microscopy. *Cell reports* **25,** 1371–1383.e10. ISSN: 2211-1247 (Oct. 2018).

18. Yamaguchi, A. *et al.* Multi-neuronal recording in unrestrained animals with all acousto-optic random-access line-scanning two-photon microscopy. *Frontiers in Neuroscience* **17.** ISSN: 1662-453X (2023).

19. Seelig, J. D. *et al.* Two-photon calcium imaging from head-fixed Drosophila during optomotor walking behavior. en. *Nature Methods* **7,** 535–540. ISSN: 1548-7091 (July 2010).

20. Seelig, J. D. & Jayaraman, V. Neural dynamics for landmark orientation and angular path integration. en. *Nature* **521,** 186–191. ISSN: 0028-0836 (May 2015).

21. Sawinski, J. *et al.* Visually evoked activity in cortical cells imaged in freely moving animals. en. *Proceedings of the National Academy of Sciences* **106,** 19557–19562. ISSN: 0027-8424, 1091-6490 (Nov. 2009).

22. Zong, W. *et al.* Fast high-resolution miniature two-photon microscopy for brain imaging in freely behaving mice. en. *Nature Methods* **14,** 713–719. ISSN: 1548-7091 (July 2017).

23. Dombeck, D. A., Harvey, C. D., Tian, L., Looger, L. L. & Tank, D. W. Functional imaging of hippocampal place cells at cellular resolution during virtual navigation. en. *Nature Neuroscience* **13,** 1433–1440. ISSN: 1097-6256 (Nov. 2010).

24. Flusberg, B. A. *et al.* High-speed, miniaturized fluorescence microscopy in freely moving mice. en. *Nature Methods* **5,** 935–938. ISSN: 1548-7091 (Nov. 2008).

25. Helmchen, F., Fee, M. S., Tank, D. W. & Denk, W. A Miniature Head-Mounted Two-Photon Microscope: High-Resolution Brain Imaging in Freely Moving Animals. *Neuron* **31,** 903–912. ISSN: 0896-6273 (Sept. 2001).

26. Maimon, G., Straw, A. D. & Dickinson, M. H. Active flight increases the gain of visual motion processing in Drosophila. en. *Nature Neuroscience* **13,** 393–399. ISSN: 1097-6256 (Mar. 2010).

27. Green, J. *et al.* A neural circuit architecture for angular integration in Drosophila. en. *Nature* **546,** 101–106. ISSN: 0028-0836 (June 2017).

28. Griffiths, V. A. *et al.* Real-time 3D movement correction for two-photon imaging in behaving animals. en. *Nature Methods* **17,** 741–748. ISSN: 1548-7091, 1548-7105 (July 2020).

29. Nadella, K. M. N. S. *et al.* Random-access scanning microscopy for 3D imaging in awake behaving animals. en. *Nature Methods* **13,** 1001–1004. ISSN: 1548-7091 (Dec. 2016).

30. Vučinić, D. & Sejnowski, T. J. A Compact Multiphoton 3D Imaging System for Recording Fast Neuronal Activity. en. *PLOS ONE* **2.** Publisher: Public Library of Science, e699. ISSN: 1932-6203 (Aug. 2007).

31. Kirkby, P. A., Srinivas Nadella, K. M. N. & Silver, R. A. A compact acousto-optic lens for 2D and 3D femtosecond based 2-photon microscopy. en. *Optics Express* **18,** 13720. ISSN: 1094-4087 (June 2010).

32. Katona, G. *et al.* Fast two-photon in vivo imaging with three-dimensional random-access scanning in large tissue volumes. *Nature Methods* **9,** 201–208. ISSN: 1548-7091, 1548-7105 (Jan. 2012).

33. Duemani Reddy, G., Kelleher, K., Fink, R. & Saggau, P. Three-dimensional random access multiphoton microscopy for functional imaging of neuronal activity. *Nature Neuroscience* **11,** 713–720. ISSN: 1097-6256 (June 2008).

34. Reddy, G. D., Cotton, R. J., Tolias, A. S. & Saggau, P. en. in *Membrane Potential Imaging in the Nervous System and Heart* (eds Canepari, M., Zecevic, D. & Bernus, O.) *Advances in Experimental Medicine and Biology* 859, 455–472 (Springer International Publishing, 2015). ISBN: 978-3-319-17640-6 978-3-319-17641-3.

35. Szalay, G. *et al.* Fast 3D Imaging of Spine, Dendritic, and Neuronal Assemblies in Behaving Animals. *Neuron* **92,** 723–738. ISSN: 0896-6273 (Nov. 2016).

36. Pereira, T. D. *et al.* SLEAP: A deep learning system for multi-animal pose tracking. en. *Nature Methods* **19.** Publisher: Nature Publishing Group, 486–495. ISSN: 1548-7105 (Apr. 2022).

Supplementary Figures

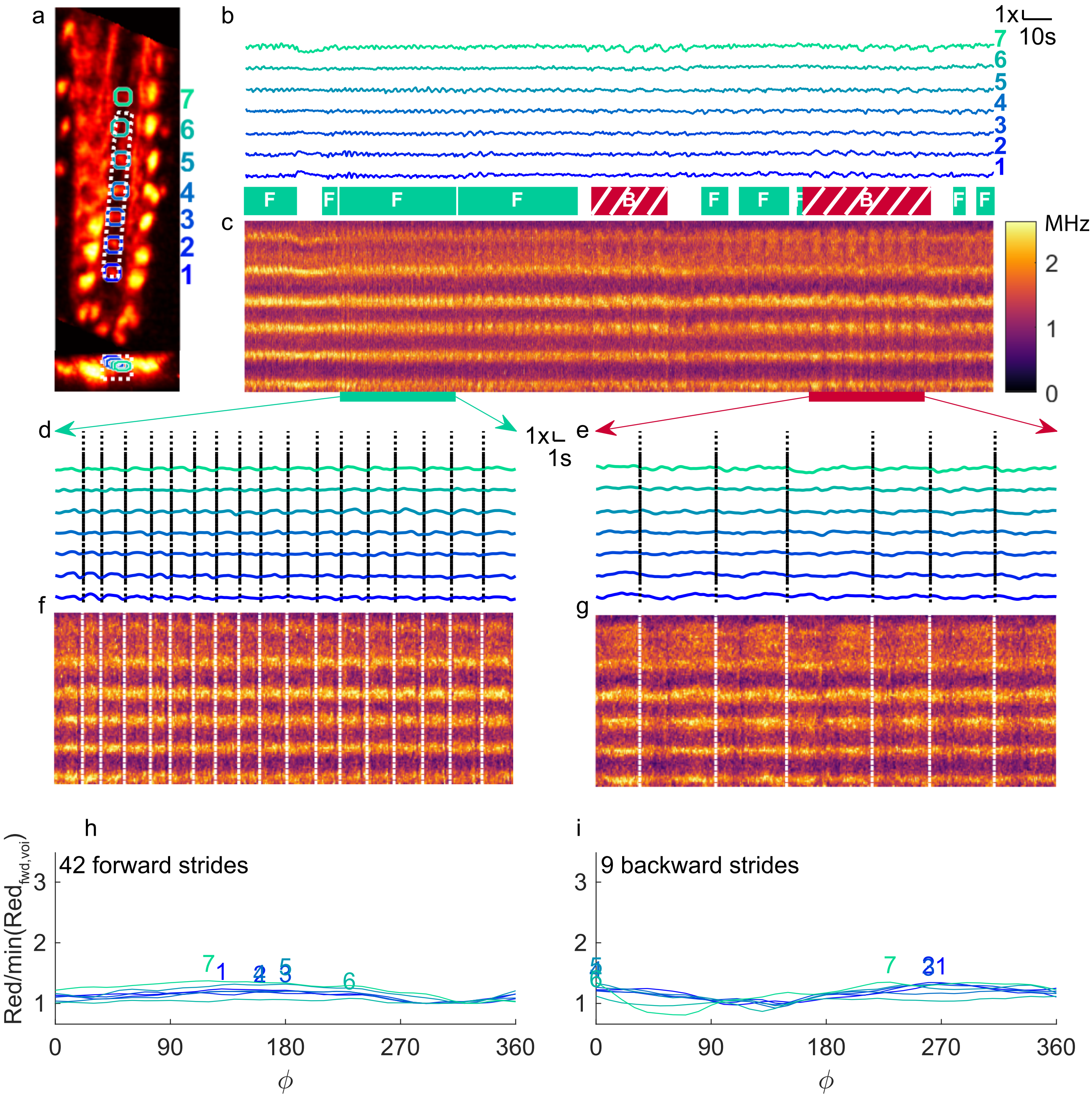

Figure S1: (Supplemental) Imaging A27h central processes - red channel control. The same analyses as in Figure 2, except the red channel is analyzed rather than the green/red ratio. In the projections (c,f,g), the rate (sum of red counts over the projected region divided by the sum of dwell time over the project region) is displayed. For this figure, we did *not* apply the intensity correction that adjusts the instantaneous red count rate to match the template, in order to show that the red signal does not contain any wave-like signals of the kind seen in the ratiometric measure.

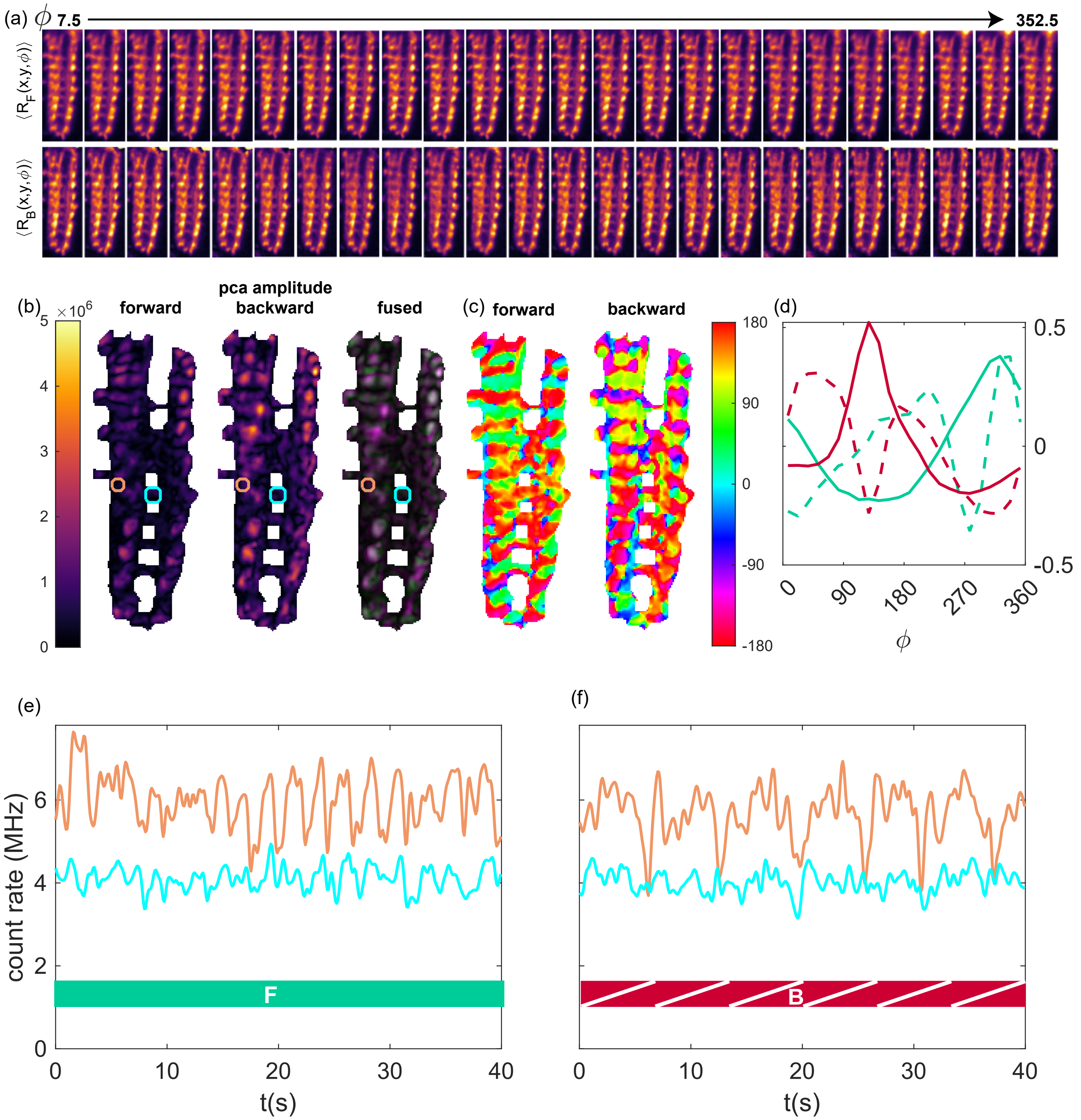

Figure S2: (Supplemental) Analysis of wave-like activity - red channel control. Same analysis as in Figure 3 but for red fluorescence (due to mCherry stable indicator) on the same data set. In (e,f) the average red fluorescence rate (rather than the normalized green/red ratiometric measure) is plotted.

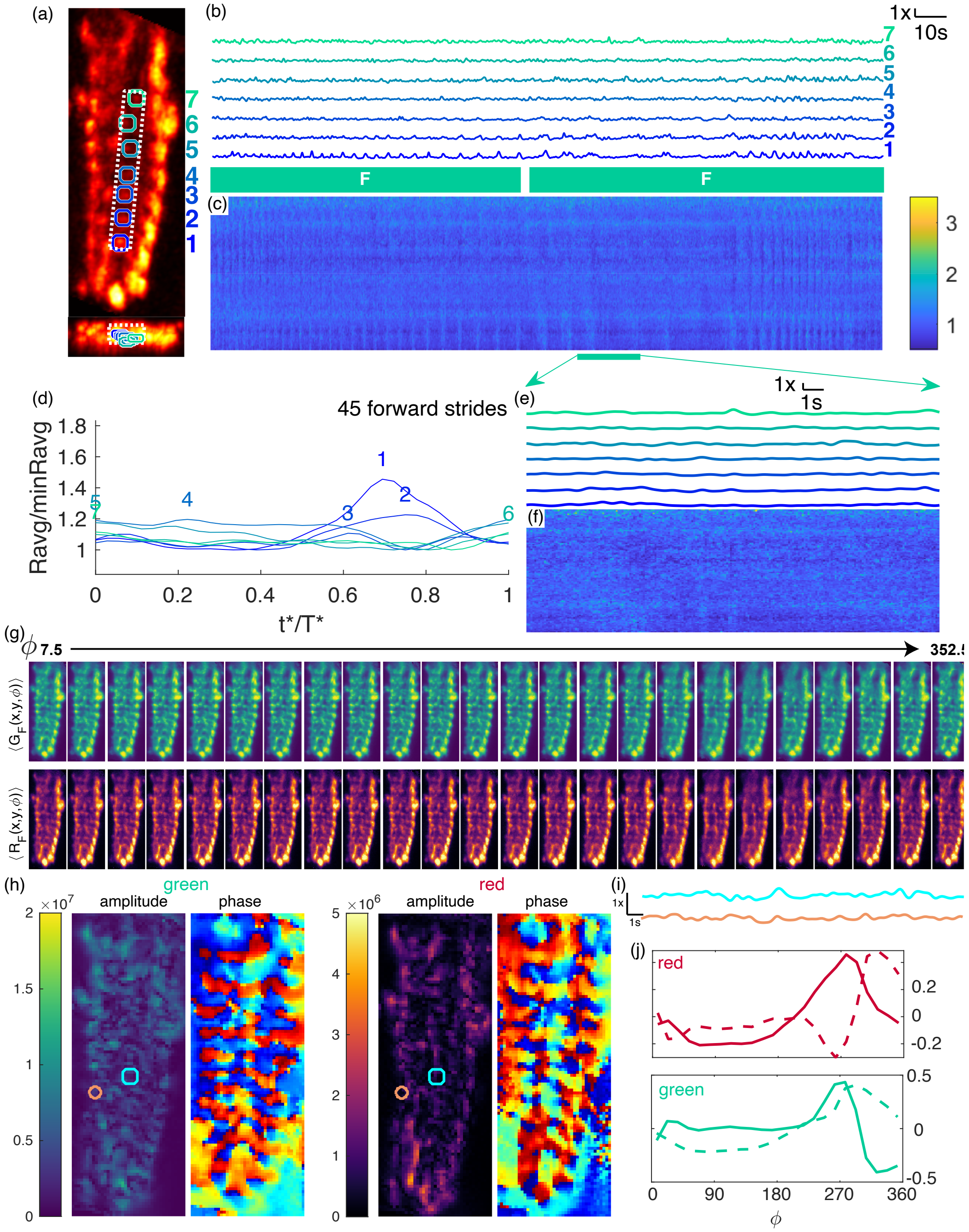

Figure S3: (Supplemental) GFP control for A27h recordings. 2nd instar larva expressed GFP and hexameric mCherry under R36G02 control. During the 213 second recording shown, the larva travelled 6 mm from its original position along a path length of 8 mm (a) X-Y and X-Z projection of template image created from mCherry channel of labeled VNC. Colored circles indicate VOIs analyzed in panels b,d,e,h,i. Vois progress numerically from posterior to anterior. White dashed line indicates volume projected in panels c,f,g (b) Ratiometric measurement of activity in VOIs over the time course of the experiment, normalized separately for each VOI. Periods of forward crawling are indicated below. Unlabeled periods represent turns and pauses. (c) Time-space projection of ratiometric activity measure. The vertical (space) axis spans the range from the bottom of the white box to the top. The horizontal (time) axis matches panel (b). The range is normalized globally so that the median of the data shown in the panel is 1. (d) Cycle-average ratiometric activity measure in each VOI for forward crawling. Each VOI is normalized separately so that the minimum mean activity is 1. (e-f) Expanded views of voi ratio and projected ratios during forward crawling. Panels c,f use the same color scale as Figure 2 (c,f,g) Note different y-axis in panel d from Figure 3h,i (g) Average green (top row) and red (bottom row) fluorescence vs. position and peristaltic phase for 45 forward strides. (h) Amplitudes and phases of first two principal component projections for red and green channels, during forward crawling. (i) Normalized ratio for two VOIs shown on (h) during time window excerpted in (e,f). (j) First two principal components for red and green channels.

.

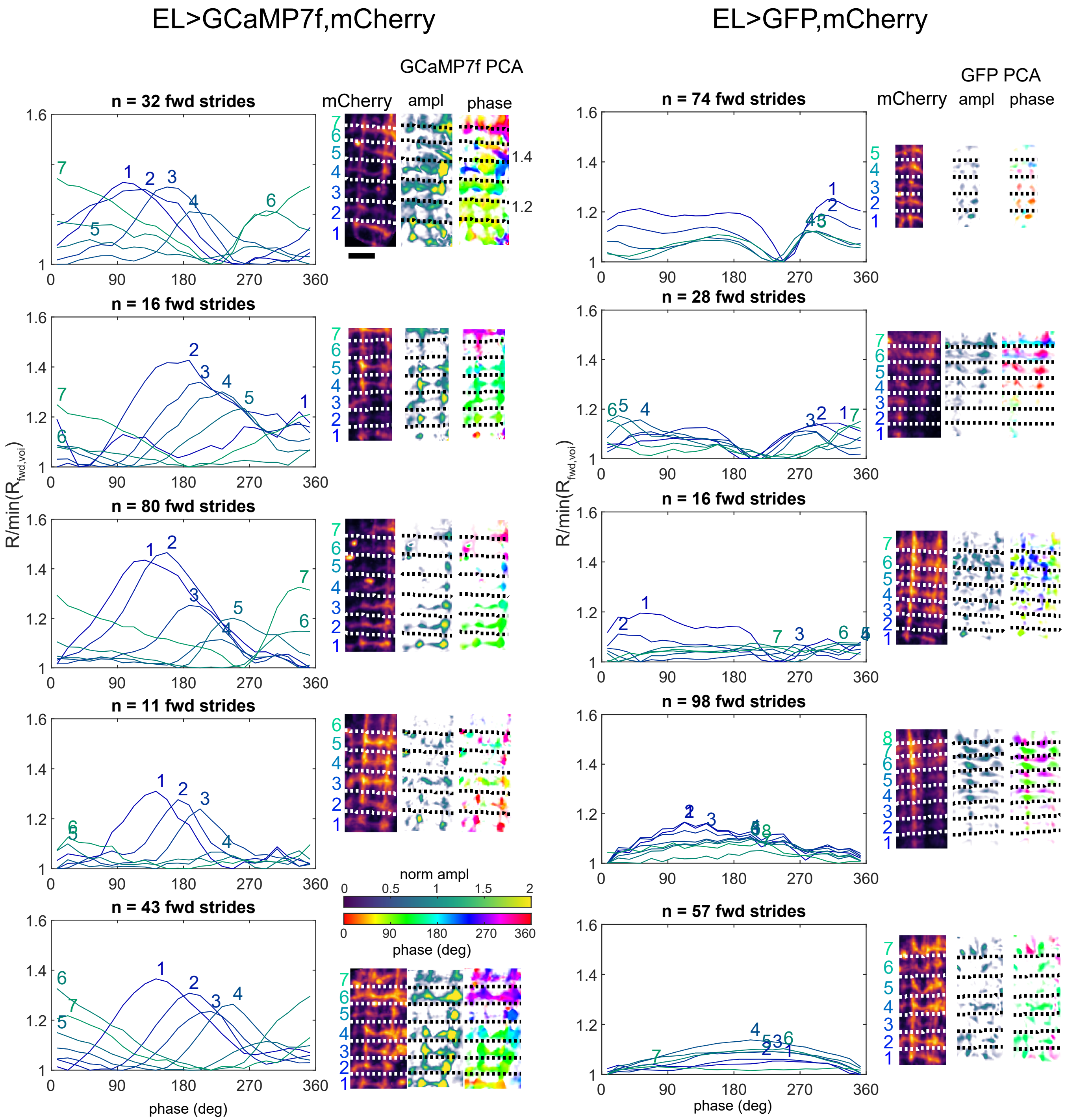

Figure S4: Supplement: Phase-correlated activity in EL processes in multiple animals. Left: Larvae expressed jGCaMP7f and mCherry under EL control. Right: Larvae expressed GFP and mCherry under EL control. Within each panel, from left to right: the stride aligned average ratio for each segment, as a function of phase, normalized so that the minimum is 1; projection of mCherry fluorescence in analyzed region, with segments indicated by dashed white lines and numbers to the left; PCA amplitude and PCA phase. Amplitude is normalized to the median green fluorescence of the imaged region. 25 micron scale bar is consistent across images.

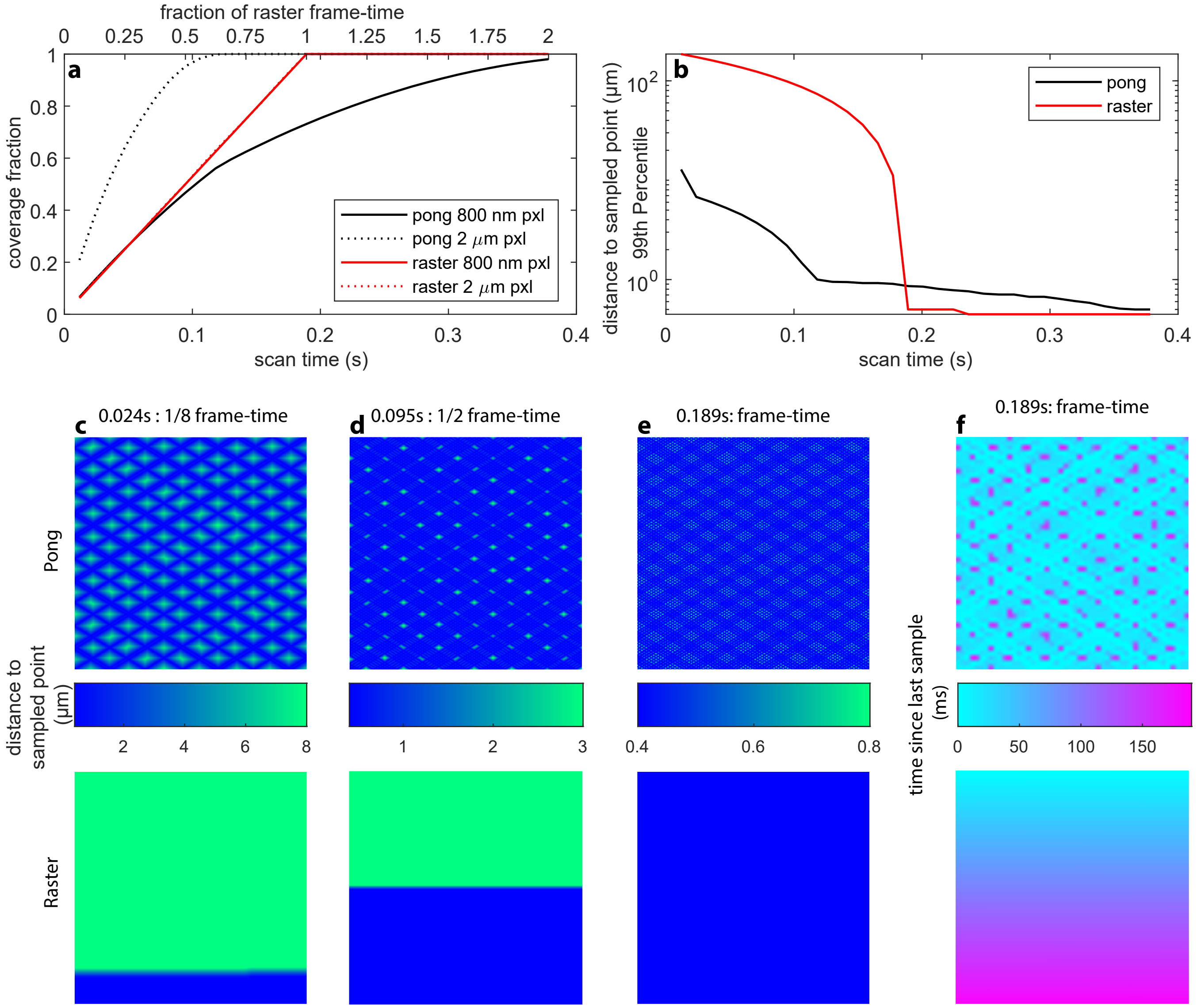

Figure S5: Supplement: sampling in pong and raster scanning. Simulated sampling of a still sample in 2D using typical experimental parameters: sampling region 200 x 200 μm, target pixel size 800 nm, bidirectional triangular x-scan at a frequency of 661 Hz (line frequency = 1322 Hz). For raster scans, the y location advanced by 800 nm after each x-line. For pong scans, there was a bidirectional triangular y-scan at a frequency of 409 Hz. The tag lens (resonant frequency: 190 kHz) produces nearly axial lines. Taking into account the lateral motion of the galvos, the central portion of the axial scan lines are displaced laterally by a maximum of 400 nm from one end to the other. The marginal effect on sampling density at the extremes of the scan is ignored in this analysis, which also ignores the $\sim$ 100 μs turnaround time at the edges of the scan. **a** The fraction of 2D pixels (3D columns) sampled at a pixel size of 800 nm and a pixel size of 2 microns vs. scan time, for both pong and raster scans. Sampling means that the focal spot passed within a pixel, not that the entire pixel was sampled. The raster frame-time, 189ms, is the time it takes a raster scan to sample the entire volume given a pixel size of 800 nm. **b** At any time, each location in the image has either been sampled or is some distance from the nearest sampled location (see panels **c-e**.). This shows the 99th percentile distance to the nearest sampled point vs. scan time. This can be interpreted as the smallest radius an object can have and be 99% likely to have been sampled at least once during the scan. Note logarithmic y-axis. **c-e** Distance to nearest sampled location, for 3 sampling durations (1/8 frame-time, 1/2 frame-time, and full frame-time), for pong (top row) and raster (bottom row) scans. The color scale indicates distance and is different for each set of images. Note that in **c,d**, the raster scan saturates the color scale - e.g. in panel **c**, most pixels are $>$ 100 μm from the nearest scanned location. **f** Time since a 4 micron square pixel at a given location was last sampled, after a 189 ms (raster frame-time) scan. As the raster proceeds from bottom to top, the bottom pixels were sampled much longer ago than the top pixels. In contrast, the pong scan distributes sampling over time more evenly. Scale: each image is 200 by 200 μm.

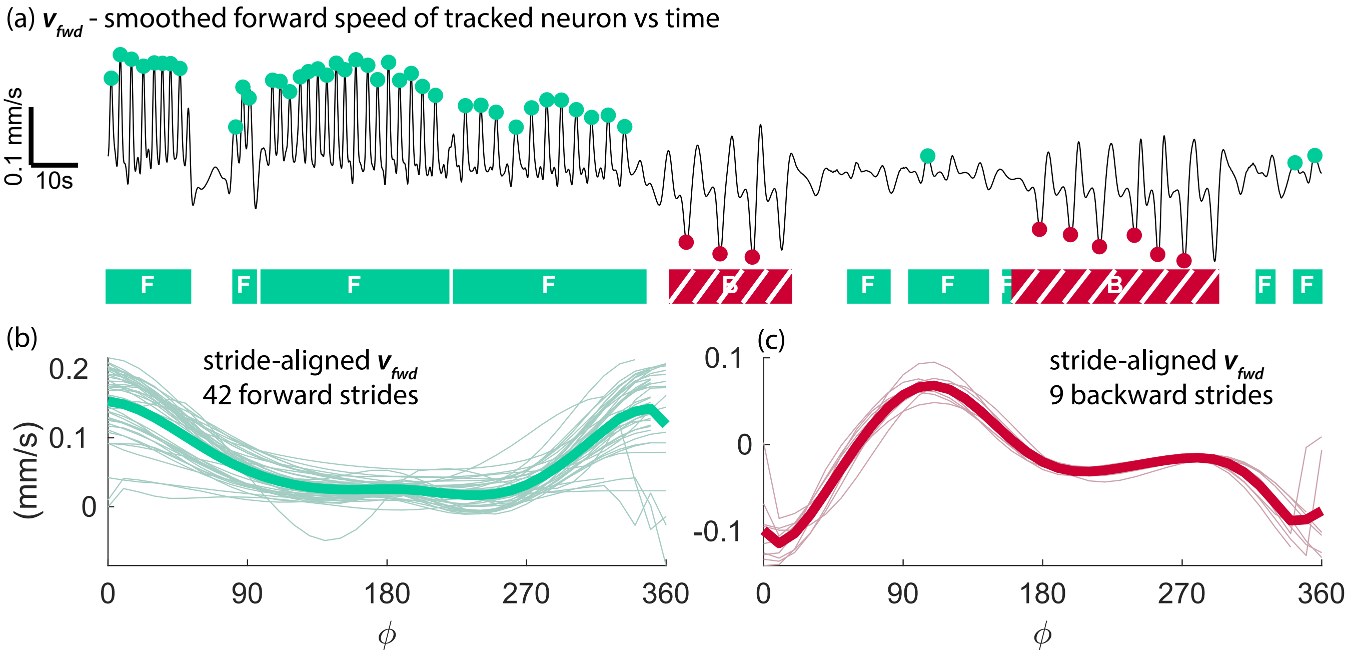

Figure S6: Supplement: Speed aligned to stride. Experiment depicted in Figure 2 (a) v_{fwd} - forward velocity used to determine the peristaltic cycle, as described in section 4.9.3. Cyan dots indicate time at which $\phi = 0$ for forward strides. Red dots indicate times at which $\phi = 0$ for backward strides. Hand-annotated behavioral states are shown below. As described in the methods, the last stride of a bout was not used, due to ambiguity of the location of $\phi = 2\pi$. (b) forward velocity aligned to peristaltic phase of forward strides following procedure described in section 4.9.4; thin lines are individual strides, thick line is the mean of all strides. (c) forward velocity aligned to peristaltic phase of backward strides following procedure described in section 4.9.4; thin lines are individual strides, thick line is the mean of all strides.

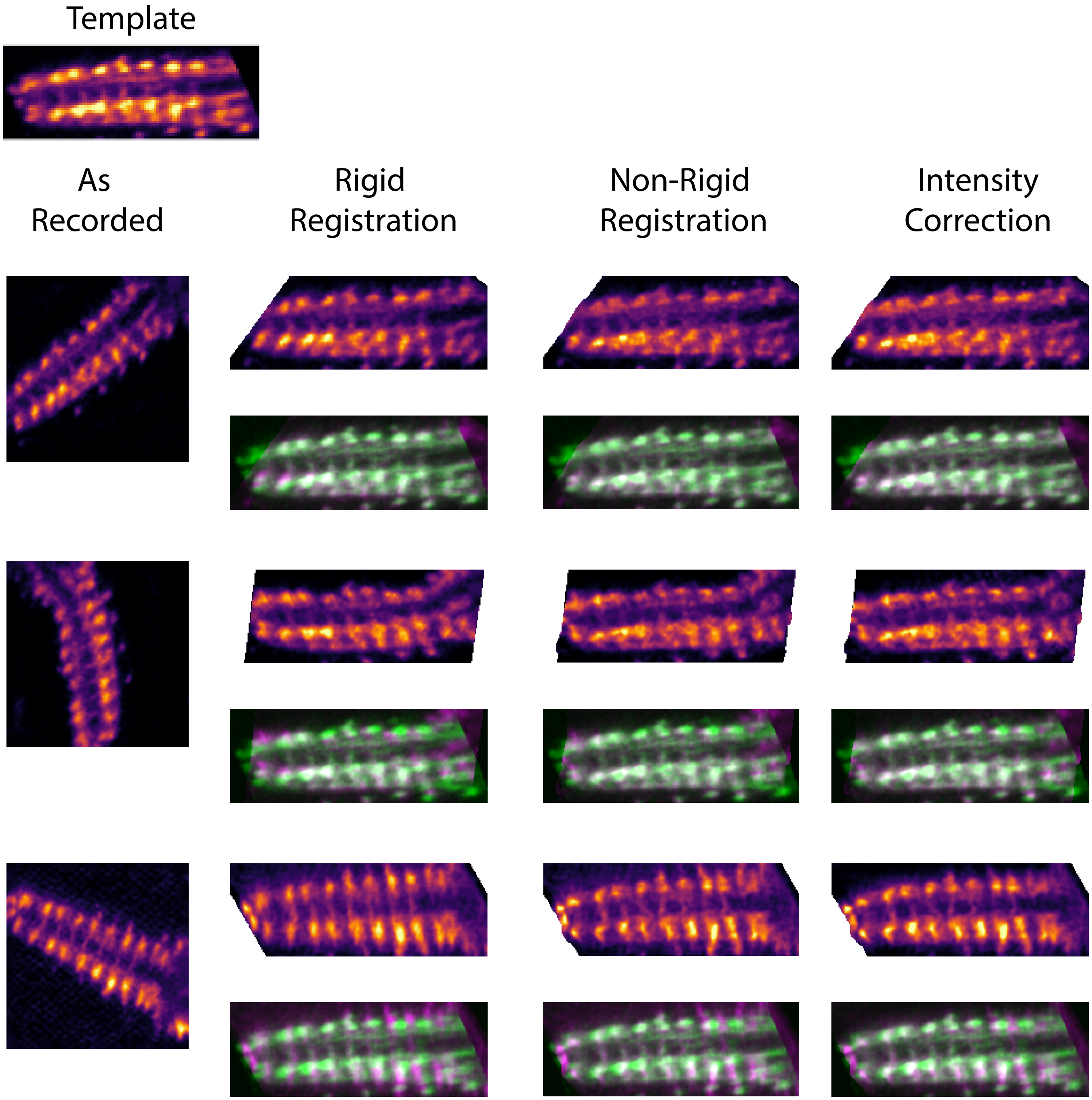

Figure S7: Supplement: registration pipeline. Experiment depicted in Figure 2 Axial projection of red (mCherry) fluorescence volumes at various stages of registration. From left to right: following acquisition, following rigid registration, following non-rigid registration, following intensity correction. Projection of the template is shown in the top left. Each registered data set is shown on its own and overlaid on the template image. For the overlaid images, the template is in green, the registered image is in purple, and the overlay is white where the two agree.

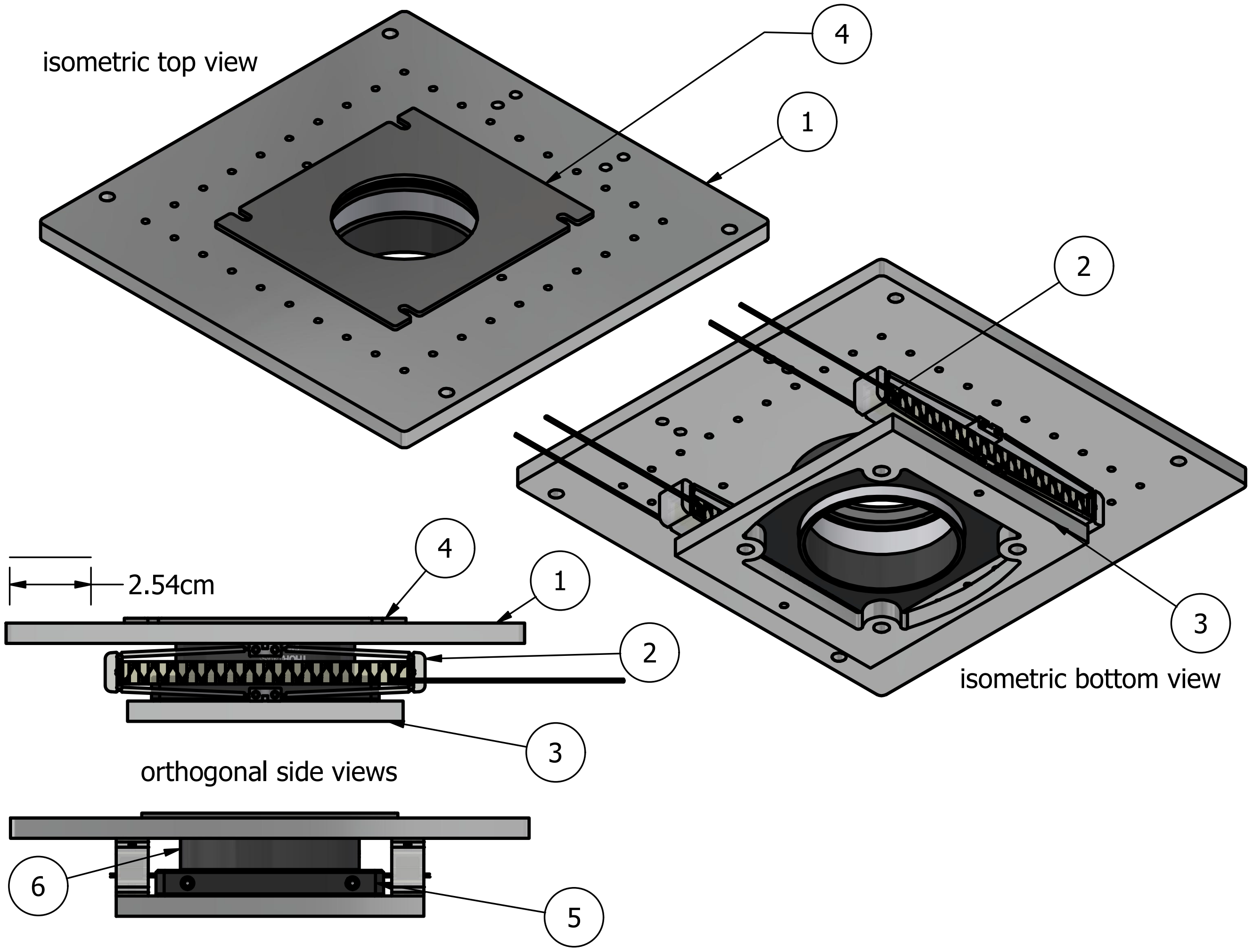

PARTS LIST			
ITEM	QTY	PART NUMBER	DESCRIPTION
1	1	top plate	laser cut from aluminum
2	2	APF710	thorlabs amplified piezo
3	1	bottom mount	laser cut from aluminum
4	1	slide holder	laser cut from steel
5	1	LCP8S	thorlabs cage plate
6	1	SM2V05	thorlabs lens tube

Figure S8: Supplement: immobilization apparatus. Major components of the immobilization device. The assembly mounted to the XYZ-stage on 0.5" optical posts (Thorlabs). A 2" diameter, 1/4"thick clear acrylic circle was laser cut to fit inside the lens tube (6) and glued to a retaining ring. The height of the acrylic above the tube was adjusted by turning the retaining ring until it barely cleared the top surface of the top plate (1) when the piezos (2) were fully contracted. With the piezos fully extended, the agarose coated coverslip containing the larva was mounted between the steel retaining plate(4) and the top plate (1). To immobilize a larva, the piezos were contracted while the larva was observed with an infrared camera, until the larva was visibly immobilized. To release the larva, the piezos were extended.
