# Supplementary material for: Closed-loop two-photon functional imaging in a freely moving animal
Source: Nat Commun. 2025 Jul 1;16:5950. doi: 10.1038/s41467-025-60648-x (PMC12214772; doi:10.1038/s41467-025-60648-x)
Supplement: Supplementary file 2 — Description of Additional Supplementary Files [file 41467_2025_60648_MOESM2_ESM.pdf]

Description of Additional Supplementary Files

Supplementary Movie 1: Calcium dynamics of A27h/M neurons in freely crawling larva. Movie shows the same larva for the same time periods as Figure 2,Figure 3. Left panel: IR image of the larva offset to account for stage motion. In the experiment, the stage moved to keep the larva centered under the objective. In this video, the stage offset it added back in, so that the total motion of the larva can be understood. The white square on the larva's body is an artifact due to the 920 nm laser penetrating the IR bandpass filter on the camera The pink dots show the approximate location of the tracked neuron at the current and each previous time point. The hand-annotated behavioral state (either forward crawl, backward crawl, or blank) is displayed in the upper right corner; the elapsed time since the start of the recording in the lower right. The top right panel shows the projected green fluorescence at the corresponding time, following the registration and intensity correction pipeline (section 4.8). The bottom right panel shows the projected red fluorescence at the same time, following registration and intensity correction. On both green and red panels, the white dashed rectangles show the bounds over which the corresponding axis was projected.

Supplementary Movie 2: Calcium dynamics of A27h/M neurons in freely crawling larva, as recorded by tracker without correction. The same data and presentation scheme as for Movie 1 but without registration or intensity correction. Note that because the VNC can be oriented arbitrarily in space, the full scan region, rather than just the subset containing cells of interest, is shown.

Supplementary Movie 3: Stride aligned calcium dynamics of A27h/M neurons in freely crawling larva. The forward and backward stride aligned mean z-projected green intensity, shown in Figure 3a, displayed as a movie, along with the stride aligned mean z-projected red intensity (Figure S2a) as a control. Note that during forward crawling, on the green channel, a wave propagates posterior (bottom)-anterior(top) in the central processes and more medial cell bodies, while during backward crawling, a wave propogates anterior-posterior along more lateral cell bodies. The sequence repeats 5 times. On the second repetition, cyan arrows indicate 12 locations where the activity is most biased towards forward crawling, according to Figure 3c, while red arrows indicate 12 locations where the activity is most biased towards backward crawling. On the third repetition, only the forward crawling (cyan) arrows are shown; on the fourth repetition, only the reverse crawling (red) arrows are shown; on the last repetition, both sets of arrows are again shown.

Supplementary Movie 4: Control GFP recording from R36G02 in freely crawling larva. Movie shows the same larva for the same time periods as Figure S3. Left panel: IR image of the larva offset to account for stage motion. In the experiment, the stage moved to keep the larva centered under the objective. In this video, the stage offset it added back in, so that the total motion of the larva can be understood. The white square on the larva's body is an artifact due to the 920 nm laser penetrating the IR bandpass filter on the camera The pink dots show the approximate location of the tracked neuron at the current and each previous time point. The hand-annotated behavioral state (either forward crawl, backward crawl, or blank) is displayed in the upper right corner; the elapsed time since the start of the recording in the lower right. The top right panel shows the projected green fluorescence at the corresponding time, following the registration and intensity correction pipeline (section 4.8). The bottom right panel shows the projected red fluorescence at the same time, following registration and intensity correction. On both green and red panels, the white dashed rectangles show the bounds over which the corresponding axis was projected.

Supplementary Movie 5: Control GFP recording from R36G02 in freely crawling larva, as recorded by tracker without correction. The same data and presentation scheme as for Movie 4 but without registration or intensity correction. Note that because the VNC can be oriented abritrarily in space, the full scan region, rather than just the subset containing cells of interest, is shown.

Supplementary Movie 6: Calcium dynamics of EL central processes in freely crawling larva. Movie shows the same larva for the same time periods as Figure 4. Left panel: IR image of the larva offset to account for stage motion. In the experiment, the stage moved to keep the larva centered under the objective. In this video, the stage offset it added back in, so that the total motion of the larva can be understood. The white square on the larva's body is an artifact due to the 920 nm laser penetrating the IR bandpass filter on the camera The pink dots show the approximate location of the tracked neuron at the current and each previous time point. The hand-annotated behavioral state (either forward crawl, backward crawl, or blank) is displayed in the upper right corner; the elapsed time since the start of the recording in the lower right. The top right panel shows the projected green fluorescence at the corresponding time, following the registration and intensity correction pipeline (section 4.8). The bottom right panel shows the projected red fluorescence at the same time, following registration and intensity correction. On both green and red panels, the white dashed rectangles show the bounds over which the corresponding axis was projected.

Supplementary Movie 7: Calcium dynamics of el neurons in freely crawling larva, as recorded by tracker without correction. The same data and presentation scheme as for Movie 6 but without registration or intensity correction. Note that because the VNC can be oriented abritrarily in space, the full scan region, rather than just the subset containing the central processes, is shown.

Supplementary Movie 8: Behavior and projected fluorescence from MDN>GCaMP6f,mcherry larva. Movie shows the same larva for the same time periods as Figure 5 trace o. Top left panel: IR image of the larva offset to account for stage motion. In the experiment, the stage moved to keep the larva centered under the objective. In this video, the stage offset it added back in, so that the total motion of the larva can be understood. The white square on the larva's body is an artifact due to the 920 nm laser penetrating the IR bandpass filter on the camera The pink dots show the approximate location of the tracked neuron at the current and each previous time point. The hand-annotated behavioral state (either forward crawl, backward crawl, or blank) is displayed in the upper right corner; the elapsed time since the start of the recording in the lower right. The bottom left panel shows the ratio of green to red fluorescence recovered from the tracked cell body (same data as in Figure 5 panel (i)) vs. time, with the current time of the video indicated by the vertical dashed line. Undershading indicates behavioral state. The top/bottom right panels show the projected green/red fluorescence at the corresponding time. A single global correction is applied equally to both images to make the temporally lowpassed fluorescence of the tracked neuron constant in time. On both green and red panels, the white dashed ellipsoids indicate the bounds of the tracked neuron used to calculate the displayed activity ratio. The bright line in both green and red XY projections running vertically from the top center to lower left in the still image above is the autofluorescent structure discussed in Section 3.1.1

Supplementary Movie 9: Behavior and projected fluorescence from MDN>GCaMP6f,mcherry larva, with projection limited to range of tracked cell body. The same as Movie 8, except the maximum intensity projection is only over the portion of the projected axis that includes the tracked cell body. For example, the XZ projection shown below the main XY panel shows the maximum value of the fluorescence intensity for a particular x and z value, over the central y-region shown by dashed lines above. This allows the tracked cell body to be seen in XZ and ZY projections despite the nearby autofluorescent structure

Supplementary Movie 10: Behavior and projected fluorescence from MDN>GFP,mcherry larva. Movie shows the same larva for the same time periods as Figure 5 trace g. Top left panel: IR image of the larva offset to account for stage motion. In the experiment, the stage moved to keep the larva centered under the objective. In this video, the stage offset it added back in, so that the total motion of the larva can be understood. The white square on the larva's body is an artifact due to the 920 nm laser penetrating the IR bandpass filter on the camera The pink dots show the approximate location of the tracked neuron at the current and each previous time point. The hand-annotated behavioral state (either forward crawl, backward crawl, or blank) is displayed in the upper right corner; the elapsed time since the start of the recording in the lower right. The bottom left panel shows the ratio of green to red fluorescence recovered from the tracked cell body (same data as in Figure 5 panel (i)) vs. time, with the current time of the video indicated by the vertical dashed line. Undershading indicates behavioral state. The top/bottom right panels show the projected green/red fluorescence at the corresponding time. A single global correction is applied equally to both images to make the temporally lowpassed fluorescence of the tracked neuron constant in time. On both green and red panels, the white dashed ellipsoids indicate the bounds of the tracked neuron used to calculate the displayed activity ratio. The faint line in both green and red XY projections visible from the center to the lower left in the still image above is the autofluorescent structure discussed in Section 3.1.1

Supplementary Movie 11: Behavior and projected fluorescence from MDN>GFP,mcherry larva, with green image adjusted to saturate at lower intensity. The same as Movie 10, except that the green image saturates at 10% of the intensity of that movie (0.5 MHz vs 5 MHz). This allows the autofluorescent structure to be seen more clearly. Note that in absolute terms, the autofluorescent structure is dimmer in the GFP recording than in the GCaMP recording (Movie 8), because the excitation laser intensity was lower (6xGFP is much brighter than GCaMP6f, so requires less excitation intensity).

Supplementary Movie 12: Registration pipeline steps. The 3 steps (rigid alignment, non-rigid alignment, and intensity correction) are demonstrated in sequence.

Supplementary Movie 13: Immobilizing and releasing a larva. A larva on an agar coated coverslip is repeatedly compressed and released using the immobilization device while recorded under infrared illumination. The larva crawls freely when not immobilized. The larva is compressed and immobilized by a piece of acrylic applied to its dorsal surface by the immobilization stage (methods 4.1.2). Note that following release from the first immobilization, a residual water droplet on the acrylic surface is visible between the larva and the camera, revealing space between the larva's dorsal surface and the acrylic. The video was recorded at 10 fps; playback at 30 fps represents 3x real time. In this demonstration video, the larva was manipulated more than in actual experiments.

Supplementary Movie 14: SLEAP labeling a larva. Larval posture was obtained through SLEAP Pereira *et al.* [36] labeling of distinct image features. Five points along a larva's body were labeled: The tip of the head, the left and right trachea, the beam square (an image artifact of our pong scan), and a gut feature. Video recorded at 10 fps; 10fps playback is real time.
