# Supplementary material for: Closed-loop two-photon functional imaging in a freely moving animal
Source: Nat Commun. 2025 Jul 1;16:5950. doi: 10.1038/s41467-025-60648-x (PMC12214772; doi:10.1038/s41467-025-60648-x)
Supplement: Supplementary file 17 — Reporting Summary [file 41467_2025_60648_MOESM17_ESM.pdf]

nature portfolio

Corresponding author(s): Marc Gershow

Last updated by author(s): Dec 5, 2024

Reporting Summary

Nature Portfolio wishes to improve the reproducibility of the work that we publish. This form provides structure for consistency and transparency in reporting. For further information on Nature Portfolio policies, see our Editorial Policies and the Editorial Policy Checklist.

Statistics

For all statistical analyses, confirm that the following items are present in the figure legend, table legend, main text, or Methods section.

n/a	Confirmed	
☐	☒	The exact sample size (*n*) for each experimental group/condition, given as a discrete number and unit of measurement
☐	☒	A statement on whether measurements were taken from distinct samples or whether the same sample was measured repeatedly
☐	☒	The statistical test(s) used AND whether they are one- or two-sided<br>*Only common tests should be described solely by name; describe more complex techniques in the Methods section.*
☒	☐	A description of all covariates tested
☒	☐	A description of any assumptions or corrections, such as tests of normality and adjustment for multiple comparisons
☐	☒	A full description of the statistical parameters including central tendency (e.g. means) or other basic estimates (e.g. regression coefficient) AND variation (e.g. standard deviation) or associated estimates of uncertainty (e.g. confidence intervals)
☐	☒	For null hypothesis testing, the test statistic (e.g. *F*, *t*, *r*) with confidence intervals, effect sizes, degrees of freedom and *P* value noted<br>*Give P values as exact values whenever suitable.*
☒	☐	For Bayesian analysis, information on the choice of priors and Markov chain Monte Carlo settings
☒	☐	For hierarchical and complex designs, identification of the appropriate level for tests and full reporting of outcomes
☒	☐	Estimates of effect sizes (e.g. Cohen's *d*, Pearson's *r*), indicating how they were calculated

Our web collection on statistics for biologists contains articles on many of the points above.

Software and code

Policy information about availability of computer code

Data collection	CRASH2p software - gershow lab development
Data analysis	MATLAB

For manuscripts utilizing custom algorithms or software that are central to the research but not yet described in published literature, software must be made available to editors and reviewers. We strongly encourage code deposition in a community repository (e.g. GitHub). See the Nature Portfolio guidelines for submitting code & software for further information.

Data

Policy information about availability of data

All manuscripts must include a data availability statement. This statement should provide the following information, where applicable:

- Accession codes, unique identifiers, or web links for publicly available datasets
- A description of any restrictions on data availability
- For clinical datasets or third party data, please ensure that the statement adheres to our policy

raw data is available through harvard dataverse -- see data availability statement

Research involving human participants, their data, or biological material

Policy information about studies with human participants or human data. See also policy information about sex, gender (identity/presentation), and sexual orientation and race, ethnicity and racism.

Reporting on sex and gender	n/a
Reporting on race, ethnicity, or other socially relevant groupings	n/a
Population characteristics	n/a
Recruitment	n/a
Ethics oversight	n/a

Note that full information on the approval of the study protocol must also be provided in the manuscript.

Field-specific reporting

Please select the one below that is the best fit for your research. If you are not sure, read the appropriate sections before making your selection.

- [x] Life sciences
- [ ] Behavioural & social sciences
- [ ] Ecological, evolutionary & environmental sciences

For a reference copy of the document with all sections, see nature.com/documents/nr-reporting-summary-flat.pdf

Life sciences study design

All studies must disclose on these points even when the disclosure is negative.

Sample size	included in figure captions and data tables<br><br>sample sizes were not pre-determined. no one has ever made two photon recordings from behaving larvae, so parameters necessary to predetermine sample size (eg magnitude of signal, magnitude and spectral properties of noise) were unknown.
Data exclusions	each experiment represents a continuous period of observation (no exclusion from within recording segments). The beginnings of experiments, when animals were still being released from immobilization , were excluded. Recordings terminated when the tracker lost the tracked neuron . Sometimes, the tracker would "jump" from the tracked neuron to a neighboring neuron. The shift of origin posed challenges to our registration pipeline, so in each experiment, we only analyzed a single period of time over which an individual neuron was continuously tracked.
Replication	a27h experiments - 10 gcamp6f labeled larvae, 5 gcamp7f labeled larvae, and 5 gfp labeled larvae were analyzed. Figure 2 and 3 show analysis of 1 of the gcamp6f experiments. Results shown are typical of the larger set of a27h>gcamp6f experiments<br>EL>GCaMP8m - figure S4 shows 5 additional experiments with EL>GCaMP7f and EL>GFP control larvae. The 8m experiment was chosen for display because the indicator provided greater contrast, but results are otherwise typical.<br>MDN experiments: 8 MDN>GCaMP6f larvae and 7 MDN>GFP larvae are presented in Figure 5<br>Each animal was used once in a separate experimental observation. n = ... strides reflects the number of forward or backward strides analyzed for that animal. all n represent real (not numerical) replicates
Randomization	Larvae of a given genotype were selected randomly from a dish of similar larvae. Selection was based on size (instar stage) only.<br><br>Determination of experimental/control groups was by genetically encoded indicator type. Calcium sensitive indicators (GCaMP family) were the experimental group; calcium insensitive indicators (GFP) were the control group.
Blinding	manual determination of behavioral state of the animal was done by investigator watching video without reference to additional neural recording data<br><br>Blinding of genotype not possible due to nature of microscopy study – genotype would be obvious to experimenter due to expression pattern and brightness of indicators.

Reporting for specific materials, systems and methods

We require information from authors about some types of materials, experimental systems and methods used in many studies. Here, indicate whether each material, system or method listed is relevant to your study. If you are not sure if a list item applies to your research, read the appropriate section before selecting a response.

Materials & experimental systems

n/a	Involved in the study
☒	☐ Antibodies
☒	☐ Eukaryotic cell lines
☒	☐ Palaeontology and archaeology
☐	☒ Animals and other organisms
☒	☐ Clinical data
☒	☐ Dual use research of concern
☒	☐ Plants

Methods

n/a	Involved in the study
☒	☐ ChIP-seq
☒	☐ Flow cytometry
☒	☐ MRI-based neuroimaging

Animals and other research organisms

Policy information about studies involving animals; ARRIVE guidelines recommended for reporting animal research, and Sex and Gender in Research

Laboratory animals	drosophila
Wild animals	n/a
Reporting on sex	due to difficulty of sexing 2nd instar larvae, experiments were performed without regard to sex. Given the nature of the study (observation of calcium dynamics of motor system command neurons, premotor interneurons, and proprioceptive interneurons), sex is not expected to influence the results
Field-collected samples	n/a
Ethics oversight	n/a

Note that full information on the approval of the study protocol must also be provided in the manuscript.

Plants

Seed stocks	*Report on the source of all seed stocks or other plant material used. If applicable, state the seed stock centre and catalogue number. If plant specimens were collected from the field, describe the collection location, date and sampling procedures.*
Novel plant genotypes	*Describe the methods by which all novel plant genotypes were produced. This includes those generated by transgenic approaches, gene editing, chemical/radiation-based mutagenesis and hybridization. For transgenic lines, describe the transformation method, the number of independent lines analyzed and the generation upon which experiments were performed. For gene-edited lines, describe the editor used, the endogenous sequence targeted for editing, the targeting guide RNA sequence (if applicable) and how the editor was applied.*
Authentication	*Describe any authentication procedures for each seed stock used or novel genotype generated. Describe any experiments used to assess the effect of a mutation and, where applicable, how potential secondary effects (e.g. second site T-DNA insertions, mosiacism, off-target gene editing) were examined.*
